# Risk Factors for Dog Relinquishment to a Los Angeles Municipal Animal Shelter

**DOI:** 10.3390/ani5040413

**Published:** 2015-12-10

**Authors:** Emily D. Dolan, Jamie Scotto, Margaret Slater, Emily Weiss

**Affiliations:** 1 Shelter Research and Development, Community Outreach, American Society for the Prevention of Cruelty to Animals (ASPCA^®^), 307 NE Thornton Place #510, Seattle, WA 98125, USA; 2 Shelter Research and Development, Community Outreach, American Society for the Prevention of Cruelty to Animals (ASPCA^®^), 520 8th Ave, New York, NY 10018, USA; E-Mail: jamie.scotto@aspca.org; 3 Shelter Research and Development, Community Outreach, American Society for the Prevention of Cruelty to Animals (ASPCA^®^), 50 Stone Ridge Drive, Florence, MA 01062, USA; E-Mail: margaret.slater@aspca.org; 4 Shelter Research and Development, Community Outreach, American Society for the Prevention of Cruelty to Animals (ASPCA^®^), 3201 SW Winding Way, Palm City, FL 34990, USA; E-Mail: emily.weiss@aspca.org

**Keywords:** pet relinquishment, animal shelter, poverty, dogs, perceived stress

## Abstract

**Simple Summary:**

Low income has been reported to be a risk factor for dog relinquishment to shelters in the U.S. The majority of people with lower incomes, however, do not relinquish. Risk factors for relinquishment in a low socioeconomic region of Los Angeles were examined. Cost was associated with relinquishment, and most people were not aware of available assistance. Those who relinquished reported emotional attachment to the dog and higher perceived stress than a comparison group. The majority of reasons for relinquishment were likely solvable with assistance, highlighting an opportunity to provide community-specific alternatives to relinquishment.

**Abstract:**

Dog relinquishment is a large component of shelter intake in the United States. Research has shown traits of the dog are associated with relinquishment as well as general characteristics of those relinquishing. Low income is often cited as a risk factor for relinquishment. The majority of people with lower incomes, however, do not relinquish. A group of people accessing a shelter in a low socioeconomic region of Los Angeles to relinquish their dogs was surveyed. This study examined risk factors for relinquishment, controlling for household income, compared to a group utilizing low cost spay/neuter services. A total of 76.9% of those relinquishing noted cost as a reason for relinquishment. Of participants in the relinquishment group, 80.7% reported not being aware of any services available to them. Most notable in the findings was that the odds of relinquishment were generally higher as the amount of perceived stress in the home in the past three months increased. The majority of people in both groups reported being emotionally attached to the dog. In this sample from a South Los Angeles community, the majority of reasons for relinquishment were likely solvable with assistance. These findings highlight an opportunity to assess community needs and provide community specific alternatives to relinquishment.

## 1. Introduction

Pet relinquishment makes up a substantial component of shelter intake across the country [1] and can account for 25%–50% of shelter intake in most communities [1,2]. As these animals were at one point in a home, the most expedient way to reduce homelessness for these animals may be to keep them at home [3]. In order to develop interventions that prevent relinquishment and keep pets home, it is important to understand the situation in the home that leads up to relinquishment and the barriers to keeping the animal. 

Much of the literature on dog relinquishment describes the characteristics of the dog that are associated with risk of relinquishment. For example, studies have reported that relinquishment is associated with the age of the dog [4,5], size of the dog [6], breed [4], whether the dog was trained [7], had a history of medical and/or behavioral issues [8], and was sick or injured [9]. Other reports show that “dogs spending most of their time outside” was associated with relinquishment of those dogs [2,10,11].

Also of importance are the numerous studies that have been published that describe general characteristics of people who relinquish dogs to animal shelters. Typically, younger adults [2,4] less than 50 years old [4], people with lower annual income (less than $20,000–$30,000) [6,10] and an educational achievement below the high school level [2,4] have been shown to be at a higher risk of relinquishing dogs. Often, the literature describes relinquishment associated with not living alone [6,10] and living in rental properties [10]. Shore, Peterson and Douglas [6] found that dog size and quantity restrictions instituted by landlords were associated with relinquishment. The sex of the owner may play a role, though the literature does not agree on whether males or females are associated with higher risk [4,5,12]. Frequently, a mismatching of the animals behavior and the expectations of appropriate behavior by the owner is associated with higher risk of relinquishment [13]. Those relinquishing pets for behavior reasons in one study [14] made misattributions or over-generalizations about the adopted animal and subsequently returned it noting behavior as the reason for relinquishment. These adopters reported that they would choose different characteristics in their next dog, perhaps indicating that their expectations had not been met. Further, in a study by Diesel [15], only half of the people said that the time and effort involved in the care of the dog was as expected. Thirty-six percent said that the time and effort they put in was more than expected.

Less is known about the actual process or events leading to relinquishment (e.g., household barriers and options considered). However, there are a few relevant studies that add to the picture of relinquishment. A study that interviewed people relinquishing dogs and cats at 12 shelters around the USA showed the top three reasons given for relinquishment were lack of time, personal problems and allergies [5]. In a study of large dog relinquishment, 68% of participants in New York City (NYC) and 98% of participants in Washington, DC (DC) said that something had changed recently in the household that contributed to the decision to relinquish the dog [16]. The most commonly cited change was related to “people issues” (e.g., health and time), followed by moving, landlord issues and behavior issues. DiGiacomo, *et al.* [17] found that time and money were key factors related to relinquishment and that the decision to relinquish animals was not sudden, lasting weeks to months for many people. Further, DiGiacomo, *et al.* [17] reported that some people attempted to resolve the issues themselves (such as attempts to re-home the dogs), but were not aware of solutions. Shore, *et al.* [6] also reported that owners attempted to re-home their dogs (often through people known to the owner). 

Conflicting evidence has been found about the role of attachment and relinquishing pets; Shore, *et al.* [6] concluded that some relinquishers had high levels of emotional involvement while Kwan and Bain [18] found less attachment. This conflict may be simply due to the wording of the question or the time at which the question was asked. DiGiacomo, *et al.* [17] found that relinquishers went through a grieving process prior to relinquishment which may explain an emotional disconnect at the time of relinquishment. 

Interestingly, factors associated with dog relinquishment have been found to vary by community. In a 2014 study conducted by Weiss *et al.* [16] the authors discovered that the frequency of the reasons for relinquishment in two large urban communities were not equal. As an example, while relinquishment in both communities cited similar reasons, a higher percentage of people relinquishing in NYC cited personal or family issues as the reason, and a higher proportion of DC people cited landlord issues as a reason. The dog and human demographics varied dramatically as well, with 44% of the relinquished dogs in NYC being reported by the owner as purebred *vs.* 5.7% in DC, and 80% of the dogs in NY being reported as a bully breed type *vs.* 57% in DC. Ethnicity, education, type of dwelling and some other factors differed as reasons for relinquishment in the two communities. This data cautions against a broad stroke approach to the development of solutions for relinquishment. 

Reasons for relinquishment can vary from community to community [16], and there is still much to be learned regarding relinquishment. This study was therefore designed to determine risk factors associated with relinquishment in a south Los Angeles municipal shelter. Low income or cost factors have been reported as factors related to relinquishment [6,10,16]; yet many low-income pet owners keep their pets. This study was restricted to an area of high poverty, and case and comparison people were from the same location. Making the two groups more similar in economic circumstances allowed the study to explore factors other than income that may be associated with relinquishment. 

The objectives were to study the following aspects in a high poverty community: (1) factors in the home that contributed to the decision to relinquish and (2) differences in demographics, attitudes and behaviors in relinquishers and their dogs and a comparison group from the same community. This information can aid policy makers and animal welfare groups in allocating resources in South Los Angeles most efficiently, in developing interventions in a targeted way to make them most applicable to the community in which they are instituted, and in developing prevention methods that may intervene with people before the decision to relinquish has been finalized. 

## 2. Experimental Section

### 2.1. Subjects

This survey was conducted June through September, 2014 at the Chesterfield Square/South Los Angeles Animal Services facility. Chesterfield Square/South L.A. is one of the six Animal Services facilities in the city of Los Angeles that are open-admission shelters and provide animal control services. Spay/neuter is mandatory in Los Angeles and proactive enforcement is conducted by animal control officers who issue notices to comply with the law. Each L.A. Animal Services facility serves a set of city zip codes. The Chesterfield Square/South L.A. shelter serves 17 zip codes and took in approximately 7165 dogs in 2014, 23% of which were relinquished by their owners. Owners are able to relinquish pets to Animal Services during open hours, Tuesday through Saturday from 8 AM to 5 PM and Sunday 11 AM to 5 PM, no appointment necessary. Owners are asked to pay a fee of $25 to relinquish their pet and provide basic information such as their name and address as well as the pet’s name, age, breed and the reason they are relinquishing the pet for Animal Services’ records. Alternatives to relinquishment are available for some pet owners entering the shelter through an on-site animal welfare group (unrelated to the shelter) whose mission is to keep pets out of shelters. The ethnicity of this area of South Los Angeles is made up of approximately 57% Hispanic or Latino people, 38% black, 2% white, 2% Asian and 2% other [19]. Renters make up 63% of the households. Only 8% of college age residents hold a college degree. This area is made up primarily of lower income households [19]. 

Data collection was conducted from 26 June 2014 through 9 September 2104. A bilingual American Society for the Prevention of Cruelty to Animals (ASPCA) interviewer was stationed at the shelter generally from Tuesday through Saturday during open shelter hours. All visitors to Animal Services were approached by the interviewer on the sidewalk, between the parking lot and the shelter itself. Two interviewers conducted all of the surveys during the time period, but only one interviewer was stationed at a time, thus some visitors may not have been approached if the interviewer was conducting a survey or on a break. The interviewer determined if the visitors were intending to relinquish a pet. The interviewer invited them to take the five to seven minute survey prior to entering the shelter and completing the relinquishment process. If a participant had more than one dog, the participant was instructed to choose one of the dogs about which to answer questions. Survey participants were informed in writing that the survey was not mandatory, they were able to stop the survey at any time, there were no direct benefits including compensation for participating in the survey, personal information would not be shared or distributed, and all answers were anonymous. This consent form was provided in English and Spanish to accommodate the high number of Spanish speakers in the service area. If the consent form was signed, the ASPCA interviewer proceeded with the survey. A total of 173 relinquishment group surveys were completed at the Chesterfield Square/South L.A. facility.

A comparison group was identified and consisted of owners from the same catchment area who were not intending to relinquish pets. The 145 comparison group participants were those who used no-cost or low-cost spay/neuter services provided to residents served by the Chesterfield Square/South L.A. Animal Services facility. The service providers were the ASPCA Spay/Neuter clinic co-located at the Chesterfield Square/South L.A. facility and Amanda Foundation mobile clinics in the South L.A. and Watts neighborhoods. An ASPCA interviewer approached all pet owners bringing in a dog or cat for spay/neuter services from 9 July 2014 through 20 September 2014. Again, only one interviewer was stationed at a time, thus some visitors may not have been approached if the interviewer was conducting a survey or on a break. The interviewer was stationed at the shelter clinic generally from Tuesday through Saturday during open clinic hours or at the mobile clinic when it was operating in the catchment area zip codes. Comparison participant surveys were conducted by the same interviewers who conducted the relinquishment survey and were provided with the same Spanish or English written consent form. 

To reduce the risk of selection bias and exposure misclassification, all participants were drawn from the same socio-geographic area during the study period. This was possible because both the shelter and the spay/neuter clinics required clients to live inside a specific catchment area (by zip code). All new cases presenting at the shelter and spay/neuter clinics during the data collection times were included. Because the study was specifically intended to include participants from the same catchment area (and the only shelter and spay/neuter clinics in that area), the comparison group would include people who would have gone to that shelter and been captured as a relinquisher if they had wanted to relinquish. 

### 2.2. Survey

The survey instrument asked 14 questions about the dog owner: whether they owned or rented their home and what type of home it was, the number of adults and children living in the home, sex, primary language in the home, age, marital and employment status, education level attained, ethnicity and race, estimated annual household income, and whether they were receiving public assistance. The survey also included another six questions regarding stress in the home (“Over the past 3 months, how would you rate the amount of stress in your home?” on a scale from 1 (no stress) to 6 (extreme stress)), to what extent they agreed they were emotionally attached to the dog, if the dog behaved the way they expected a dog to behave, whether the dog was more work to care for than they thought it would be, and what types of assistance they had tried to obtain or knew to be available in their community. Another 20 questions asked about the dog: name, species, primary caregiver, how long they had the pet, breed, size, sex and spay/neuter status, age, had the dog ever had a litter, was it microchipped, had it received a rabies vaccine or been to the vet, where they had gotten the dog, how many other animals they had, where the dog had primarily lived, whether there was adequate transportation available to care for the dog, if anything had changed in the household recently that contributed to the decision to bring the dog to the shelter, the dog’s body condition score, and the owner’s reasons for relinquishment. The comparison group was not asked the questions regarding reason for relinquishment. The comparison group was asked, instead, if they had considered relinquishment in the last month.

Surveys were conducted as averbal, face-to-face interview, and responses were recorded by the ASPCA interviewer. Two trained interviewers were used over the course of the study period to minimize interviewer bias. Both interviewers were bi-lingual English and Spanish speakers in order to provide questions in the language most comfortable for the survey taker. Following the survey, relinquishers were asked if they would like to speak to a counselor with the on-site welfare group providing assistance to try to help them keep their dog or, if they chose not to speak with the counselor, they were directed to the location where they could relinquish their dog. The choice to accept assistance or relinquish was recorded. Relinquishers were not aware that assistance would be offered prior to participation in the study. Comparison group owners who had been referred to the spay/neuter clinic by the welfare group counselor previously were not eligible for participation to reduce the risk of misclassification. 

A total of 318 surveys (173 relinquishers and 145 comparison owners) were conducted. This study originally intended to examine people relinquishing cats as well as dogs. Only six people intending to relinquish cats (and 19 comparisons) were surveyed, so people relinquishing cats were excluded from the analysis. Two people who completed fewer than 20% of the questions were excluded. This yielded a total of 291 people with dogs who completed a survey, of which 166 were in the relinquishment group and 125 in the comparison group. 

### 2.3. Statistical Analysis

Characteristics of the relinquishment and comparison groups were described using frequencies and percentages. A rank sum test was used to assess the significance of the relinquishment and comparison groups for perceived stress, number of adults, children and other pets in the household (these continuous variables were not found to be normally distributed). A t-test assuming unequal variance was used to compare the mean body condition scores of the pets between the two groups. Univariable associations were assessed between the outcome (relinquishment/non-relinquishment) and the independent variables using chi square tests for independence (significant associations were considered *p* < 0.05). To identify demographic, attitudinal and behavioral risk factors, multiple logistic regression was conducted with the outcome of relinquishment or comparison status using Stata/IC 13.1 (StataCorp LP, College Station, TX, USA). Factors determined to be associated with the outcome were added into the model and a significant likelihood ratio test (*p* < 0.05) was used to determine inclusion of each factor into a final model. 

## 3. Results 

### 3.1. Participants

Based upon the number of people approached who met the inclusion criteria, this study had approximately a 68% response rate in the relinquishment group and approximately a 75% rate in the comparison group (these numbers are approximate because there were three days at the beginning of the study where the interviewers did not write down the number of people who declined). The reasons stated for not wanting to participate were most frequently that they did not have time or they did not want to participate. 

See Table 1 for descriptive information on the relinquisher and comparison groups. 

**Table 1:** Participant characteristics and their attitudinal and behavioral responses.

Variable	Relinquishers N = 166 N (%)	Comparisons N = 125 N (%)	*p*-Value
Race *			
Hispanic or Latino	124 (74.7)	109 (87.2)	
Black or African American, non-Hispanic	30 (18.1)	14 (11.2)	
White, non-Hispanic	4 (2.4)	8 (6.4)	
Asian or Pacific Islander	2 (1.2)	3 (2.4)	
American Indian/Alaska Native	2 (1.2)	1 (0.8)	
Employment status			0.3
FT	30 (18.1)	28 (22.4)	
PT	22 (13.3)	36 (28.8)	
Student/retired	11 (6.6)	16 (12.8)	
Unemployed	41 (24.7)	38 (30.4)	
Refused/missing	62 (37.4)	7 (5.6)	
Marital status			0.9
Divorced	14 (8. 4)	12 (9.6)	
Married	68 (41.0)	54 (43.2)	
Single	80 (48.2)	58 (46.4)	
Refused/missing	4 (2.4)	1 (0.8)	
Sex			0.002
Female	108 (65.1)	99 (79.2)	
Male	57 (34.3)	22 (17.6)	
Refused/Missing	1 (0.6)	4 (3.2)	
Age (years)			0.5
18–29	35 (21.1)	25 (20.0)	
30–39	33 (19.9)	22 (17.6)	
40–49	46 (27.7)	27 (21.6)	
50–59	35 (21.1)	36 (28.8)	
60+	17 (10.2)	14 (11.2)	
Refused/missing	0 (0.0)	1 (0.8)	
Income ($)			0.8
<10 k	56 (33.7)	35 (28.0)	
10–14,999	25 (15.1)	16 (12.8)	
15–19,999	19 (11.5)	12 (9.6)	
20–24,999	16 (9.6)	15 (12.0)	
25–29,999	13 (7.8)	14 (11.2)	
30+	21 (12.7)	18 (14.4)	
Refused/Missing/Other	14(8.4)	15(12.0)	
Don’t know	2 (1.2)	0 (0.0)	
Rent or Own			0.003
Rent	125 (75.3)	80 (64.0)	
Own	29 (17.5)	42 (33.6)	
Refused/Missing	12 (7.2)	3 (2.4)	
On Public Assistance			0.002
Yes	65 (39.2)	28 (22.4)	
No	97 (58.4)	96 (76.8)	
Refused/Missing	4 (2.4)	1 (0.8)	
Education			0.004
8th grade or less	23 (13.9)	32 (25.6)	
9–12 grade	32 (19.3)	16 (12.8)	
HS diploma/GED	55 (33.1)	26 (20.8)	
Some college	29 (17.5)	29 (23.2)	
More than college	14 (8.4)	20 (16.0)	
Refused/missing	13 (7.8)	2 (1.6)	
Primary language			0.03
Spanish	80 (48.2)	74 (59.2)	
English	45 (27.1)	28 (22.4)	
Other/multi	7 (4.2)	16 (12.8)	
Refused/missing	34 (20.5)	7 (5.6)	
Type of house			0.04
Single family	82 (49.4)	83 (66.4)	
Not-single family	63 (38.0)	38 (30.4)	
Refused/missing/other	21 (12.7)	4 (3.2)	
Access to transportation			0.2
Yes	154 (92.8)	111 (88.8)	
No	11 (6.6)	14 (11.2)	
Refused/missing	1 (0.6)	0 (0.0)	
Perceived stress Level			0.000
1 No stress	22 (13.3)	52 (41.6)	
2	18 (10.8)	27 (21.6)	
3	48 (28.9)	23 (18.4)	
4	40 (24.1)	12 (9.6)	
5 & 6 High stress	37 (22.3)	11 (8.8)	
Refused/missing	1 (0.6)	0 (0.0)	
Emotionally attached to dog			0.001
Disagree/strongly disagree	0 (0.0)	0 (0.0)	
Neutral	32 (19.3)	10 (8.0)	
Agree	21 (12.7)	34 (27.2)	
Strongly agree	112 (67.5)	81 (64.8)	****
Refused/Missing	1 (0.6)	0 (0.0)	****
Dog behaves the way I expected a dog to behave			0.000
Neutral	68 (41.0)	26 (20.8)	****
Agree	39 (23.5)	40 (32.0)	****
Strongly agree	50 (30.1)	59 (47.2)	****
Refused/Missing	9 (5.4)	0 (0.0)	****
Dog is more work to care for than expected			0.000
Disagree	40 (24.1)	58 (46.4)	****
Neutral	99 (59.6)	46 (36.8)	****
Agree	26 (15.7)	21 (16.8)	****
Refused/Missing	1 (0.6)	0 (0.0)	****
Dog’s Primary Caregiver			0.07
Yes	158 (95.2)	113 (90.4)	
No	7 (4.2)	12 (9.6)	
Refused/Missing	1(0.6)	0(0.0)	
Median number of adults in the house (range)	2 (1–7)	2 (1–10)	0.02
Median number of children in the house (range)	1 (0–5)	1 (0–8)	0.6
Median number of other pets still in the home (range)	0 (0–10)	1 (0–18)	<0.001
Median perceived stress in the home over the past 3 months	3	2	<0.001

Refused, missing, other and don’t know responses were not included when performing the χ^2^test; * Participants could choose more than one response for race, so percentages may not equal 100% and chi-squared statistics are not calculated.

See Table 2 for details on the dogs in the study. 

**Table 2:** Distribution of responses for key factors specific to the animal being brought to the shelter.

Variable	Relinquishers N = 166 N (%)	Comparisons N = 125 N (%)	*p*-Value
Where did you get this dog?			0.03 ₸
Friend/Family/Neighbor	92 (55.4)	79 (63.2)	
Stray	35 (21.1)	18 (14.4)	
Litter of another dog	14 (8.4)	10 (8.0)	
Shelter or rescue	11 (6.6)	3 (2.4)	
Breeder	7 (4.2)	1 (0.8)	
Online or newspaper	3 (1.8)	4 (3.2)	
Pet store	1 (0.6)	2 (1.6)	
Other	2 (1.2)	8 (6.4)	
Missing	1 (0.6)	0 (0.0)	
Where is dog living?			0.02
Indoor only	71 (42.8)	56 (44.8)	
Outdoor only	52 (31.3)	23 (18.4)	
Indoor/outdoor	42 (25.3)	45 (36.0)	
Missing/other	1 (0.6)	1 (0.8)	
Dog’s age			<0.001
<12 months	40 (24.1)	40 (32.0)	
1–5 years	74 (44.6)	70 (56.0)	
6+ years	52 (31.3)	11 (8.8)	
Refused/Missing	0 (0.0)	2 (1.6)	
Don’t know	0 (0.0)	2 (1.6)	
Dog size			0.7
Small	70 (42.2)	54 (43.2)	
Medium	58 (34.9)	35 (28.0)	
Large	31 (18.7)	23 (18.4)	
Refused/Missing	7 (4.2)	13 (10.4)	
Dog’s sex			0.6
Female	85 (51.2)	60 (48.0)	
Male	81 (48.8)	65 (52.0)	
Ever been to veterinarian			0.9
Yes	92 (55.4)	74 (59.2)	
No	53 (31.9)	44 (35.2)	
Refused/Missing	0 (0.0)	1 (0.8)	
Don’t know	21 (12.7)	6 (4.8)	
Ever received rabies shot			0.3
Yes	84 (50.6)	77 (61.6)	
No	53 (31.9)	36 (28.8)	
Don’t know	29 (17.5)	12 (9.6)	
Microchipped			0.2
Yes	35 (21.1)	35 (28.0)	
No	123 (74.1)	84 (67.2)	
Don’t know	8 (4.8)	6 (4.8)	
Ever had litter			0.7
Yes	25 (15.1)	17 (13.6)	
No	134 (80.7)	104 (83.2)	
Don’t know	7 (4.2)	4 (3.2)	
Mean body condition score	4.9	5.0	0.5

Refused, missing, other and don’t know responses were not included when performing the χ^2^ test; ₸ Fisher’s exact test used if the expected value of any of the cells is fewer than 5.

### 3.2. Factors Related to Relinquishment

Table 3 shows the relinquishers’ reasons for relinquishing their dogs. Their answers were open-ended and they could indicate as many reasons as they wished. Among the 162 participants who responded, 115 (71%) stated either primarily or secondarily that cost (*i.e*., inability to pay for some care) was a factor in their decision. Specifically medical issues and being given a notice to comply with the mandatory spay/neuter law were frequently mentioned with cost as reasons for relinquishment.

**Table 3:** Reasons for relinquishment; combined primary and secondary reasons given.

Reason for relinquishment; N=162	Frequency	Percent
Cost only, no other reason given	23	14.2
Cost mentioned in conjunction with medical issues, spay/neuter or notice to comply		
Medical issues	46	28.4
Notices to comply	19	11.7
Spay/neuter services without mention of notice to comply	3	1.9
Cost mentioned in conjunction with a another reason		
Housing, e.g., pet deposit	10	6.2
Behavior, e.g., chewing	4	2.5
Other	10	6.2
Housing, without mention of cost	26	16.0
Behavior, without mention of cost	8	4.9
Medical issues, without mention of cost	6	3.7
Other, without mention of cost	7	4.3

**Figure 1:**
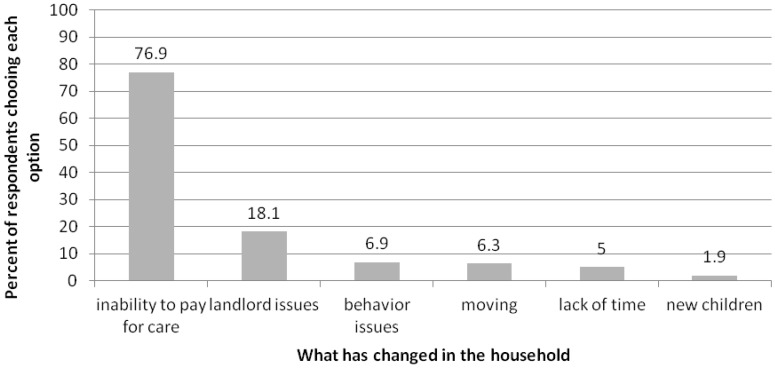
Reported changes in household prior to considering relinquishment. Participants could select more than one choice.

There were 160 relinquishers (96.0% of those who answered the question) who indicated something in the household had changed prior to relinquishment. Inability to pay for care was by far the most common response (see Figure 1). When asked if they had done anything to re-home the dog, 139 (83.7%) participants reported that going to the shelter was the first thing they tried. Twenty-five (15.1%) asked friends or family, four (2.4%) had contacted an animal rescue, two (1.2%) had received help from a shelter, and one each reported posting online, hanging flyers in public and taking the dog to public places. When the relinquishment group was given the choice of proceeding to the shelter after completing the survey or pursuing information about services that could help them keep their dog, 146 (88.0%) chose to pursue services. Only 17 (10.2%) participants proceeded to surrender their dog and three (1.8%) participants did not make a decision and left the shelter with their dog. Due to the anonymity of the survey, follow-up to see whether or not the dogs were eventually relinquished was not conducted on the participants choosing to pursue assistance. 

When asked if they knew of any support services available in their community for their dog, 134 of the relinquishment group reported not being aware of any services available to them (80.7%). Further, 26 reported being aware of free spay/neuter services (15.7%) and 15 reported being aware of free or low cost vaccination (9.0%). Only three of the relinquishment group reported being aware of safety net services co-located on the shelter grounds (1.8%). These services are provided by a private organization offering financial and other assistance to help keep animals in their homes. Only 12 of the relinquishment group were aware of free or low cost veterinary services (7.2%) and one of the relinquishment group knew about free behavioral training (0.6%). 

### 3.3. Comparisons between the Groups 

Table 1 and Table 2 show the univariable p-values when comparing the relinquisher and comparison groups.

While most dogs (117, 70.5%) in the relinquishment group were not altered, all dogs in the comparison group were recorded as altered because they were surveyed at a free spay/neuter clinic. 

Table 4 shows the results of the final multiple logistic regression model. Controlling for other factors, the odds of relinquishment were generally higher as the amount of perceived stress in the home in the past three months increased. The estimated odds of relinquishment were generally lower when participants reported being attached to the dog. Controlling for the other factors, there was no significant association between relinquishment and whether the dog behaved as expected, the dog was more work than expected, and where the dog lived. The odds were two times higher when being on public assistance. 

**Table 4:** Multiple logistic regression model of risk factors for relinquishment.

Variable	Adjusted OR (CI)	*p*-Value
Perceived stress		
1 No stress	ref	
2	1.3 (0.5–3.7)	0.6
3	4.5 (1.7–12.0)	<0.01
4	6.2 (2.0–19.5)	<0.01
5 & 6 High stress	4.9 (1.6–15.0)	<0.01
Feels emotionally attached to dog		
Neutral	ref	
Agree	0.2 (0.0–0.8)	0.03
Strongly agree	0.4 (0.1–1.6)	0.2
Dog behaves the way I expected		
Neutral	ref	
Agree	0.5 (0.2–1.3)	0.1
Strongly agree	0.6 (0.2–1.7)	0.2
Dog more work than expected		
Disagree	ref	
Neutral	1.9 (0.8–4.2)	0.1
Agree	1.7 (0.6–4.7)	0.3
Public assistance		
No	ref	
Yes	2.3 (1.1–4.9)	<0.05
Education		
8th grade or less	ref	
9–12 grade	4.4 (1.5–12.9)	<0.01
HS diploma/GED	3.6 (1.3–9.6)	<0.05
Some college	1.9 (0.7–5.5)	0.2
More than college	2.1 (0.6–6.9)	0.2
Clients’ sex		
Male	ref	
Female	0.3 (0.1–0.6)	<0.01
Where is dog living		
Indoor only	ref	
Outdoor only	1.7(0.7–4.0)	0.3
Indoor/outdoor	0.8 (0.4–1.8)	0.6
Number of other animals	0.8¥ (0.6–1.0)	0.02
Dogs’ age		
<12 months	ref	
1–5 years	1.6 (0.7–3.5)	0.2
>6 years	6.8 (2.2–21.2)	<0.01

¥ Odds ratio per each additional animal.

## 4. Discussion

This study aimed to explore the risk factors for relinquishment in a geographically-based sample drawn from a community marked by concentrated poverty. Cost alone can be associated with relinquishment, but many of low-income status still retain their pets. Thus, determining the risk factors for relinquishment within a low-income environment could provide valuable information to help further reduce relinquishment among owners in these types of locations. According to the United States census bureau, 22% of people in Los Angeles in 2009–2013 were living below the poverty line [20]. The area of South Los Angeles in particular is marked by clusters of neighborhoods with concentrated poverty, defined as more than 40% of households living in poverty [21]. Often areas of dense poverty are also resource deserts and may not receive public education and awareness messages or resources to help care for dogs. In this sample of participants, 84%–86% reported a total household income below $30,000 per year. Based on the United States Census Bureau guidelines [22], approximately 72%–74% of the participants were living in poverty. 

### 4.1. Factors Related to Relinquishment

In general, the study found that, in this population, the reported reasons for relinquishment were often potentially solvable problems. With some assistance, many of these animals could have remained in their homes. In fact, preliminary work in another shelter in LA County is beginning to show that providing assistance at the shelter door to potential relinquishers can help keep dogs home (unpublished data). Cost, and notably medical costs for veterinary concerns, was a frequently mentioned factor for relinquishment in this sample. This finding is in keeping with DiGiacomo who reported that respondents were concerned about costs [17]. Further, an inability to pay for the dogs’ needs was commonly cited as something that has changed in the household that contributed to the decision to relinquish the dog. This is similar to the findings of Weiss *et al.* [16] where participants were asked if there was any help that could have kept the dog home and providing low-cost medical care was the primary answer, though that study did not find cost of care cited frequently as a reason for relinquishment. 

Most in the relinquishment group reported not knowing about ways to get help for their dog, with the shelter being the first and only solution they had sought. This is in contrast to DiGiacomo [17], however, who concluded that people saw the shelter as a last resort and had already made up their mind when approaching the shelter. In this sample, not only was the shelter often reported as the first thing the owners tried but also most relinquishers were willing to pursue assistance before deciding to relinquish. Due to mandatory spay/neuter and other characteristics of this catchment area, relinquishers may be motivated by different forces than in other communities. This lends support to the idea that any intervention would need to be specific to meet the needs of the community. 

This study found certain differences in this geographic sample from other samples in the literature. One difference was regarding the reasons for relinquishment. For example, one study [15] showed that problematic behaviors and not being able to pay enough attention to the dog were the primary reasons for relinquishment across 14 geographically dispersed shelters in the United Kingdom. Another example of differences can be seen in a recent study of large dog relinquishment in NYC and DC [16] that found that people reported having explored many options before relinquishing their dog, with those in NY seeking more options than those in DC. In the present study, it was reported that coming to the shelter was the first thing they tried. DiGiacomo *et al.* [17] found that people tried to solve problems themselves, like re-homing their dog, but did not know how to find solutions. In this study, very few participants attempted solutions like re-homing. One explanation may be related to the wording of the questions or the selection of people for inclusion in the study. Weiss *et al.* [16], however, also noted differences regarding the populations of people relinquishing, the dogs themselves and the reasons for relinquishment between NYC and DC. It may be that populations utilizing the shelter as a re-homing option vary community by community. 

### 4.2. Comparisons between the Groups

One of the most compelling findings was that higher perceived stress in the previous 3 months within the household was found to be a significant risk factor for relinquishment when controlling for other factors. The authors are aware of no other studies that attempted to assess perceived-stress as a risk factor for dog relinquishment. It is interesting to note that Salmon *et al.* excluded 2.6% of the owners in that study because of emotional stress [2]. Learning more about stress in the home, either resulting from the pet’s situation or from another source, may be a useful target for future study. 

No one reported a lack of attachment to the animal, even in the relinquishment group. In fact, the majority reported feeling strongly attached to the dog. This is consistent with Shore’s finding that many relinquishers have high levels of emotional involvement [6]. Not surprisingly, compared to feeling neutrally about the animal, attachment was revealed to be protective. This may be explained in that relinquishing has been shown to be a difficult emotional process [10,16,17]. The findings of strong attachment and higher perceived stress in the home over the past 3 months relate to the idea that people’s attachment to their dogs makes the process of relinquishment difficult. In the DiGiacamo [17] and the Weiss [16] studies, many people thought about their decision to relinquish for a month or more. This idea is bolstered by the finding that almost every person relinquishing in this study chose to learn more about the help that could be provided when they heard about it as an alternative to relinquishment. 

It is important to note that income was not a risk factor in this study as was intended. Nor was employment a risk factor; this is likely due to the fact that there was little variation between the groups. However, because the odds of relinquishment were significantly greater when being on public assistance, there may be unmeasured external risk factors contributing to the decision to relinquish based on socioeconomic status. One such unmeasured factor may relate to immigration status. One possible explanation is that resident dog owners who are not U.S. citizens may be less likely to access the shelter as a resource. 

Controlling for the other potential risk factors, there was no significant association between relinquishment and whether the dog behaved as expected, or if the dog was more work than expected. Given that higher rates of relinquishment may be associated with unrealistic expectations of an animal [3,13], it seems likely in this study that there are other, stronger forces contributing to the decision to relinquish. For at least some participants in this study, the mandatory spay/neuter ordinance led to people coming to the shelter. And possibly related to this ordinance, financial issues the owners could not resolve alone were major reasons for relinquishment. These differences found in the South Los Angeles sample suggest that factors associated with relinquishment can be community specific, with each community having its own profile of needs. Thus solutions and interventions for relinquishment are also likely to be community specific. 

There was no strong evidence that dog characteristics were risk factors for relinquishment in this study. Factors such as size and sex were not significantly associated with relinquishment and were not significant in the multiple variable model. This differs substantially from the literature, where animal risk factors are frequently cited [4,5,6,7,8,9,10,11]. This discrepancy may largely be explained by the specific circumstances of the study community. Outside factors, such as the mandatory spay/neuter laws and cost issues as a reason for relinquishment may have contributed to relinquishment in people who were otherwise happy with the characteristics of their dogs. There was no significant relationship between relinquishment and whether the dog was living inside or outside in the multiple variable model once the other factors were accounted for. This may have important implications for animal welfare professionals who may assume that animals living outside are more likely to be relinquished to the shelter. 

A difference between previous studies and this one is its geographically-based comparison group. This comparison group allowed us to look for differences from a group of people who did not relinquish their dogs and who lived in the same geographic region, presumably facing many of the same challenges as those who had relinquished their dog. Though this study is not able to demonstrate causal relationships, the design has helped us test multiple potential risk factors for relinquishment in this community in South Los Angeles. A limitation to this study is that there was no follow up with those who pursued the available support to see if they retained their pet. The authors have, however, followed up with a group of potential relinquishers from another shelter in LA County and found that the majority contacted had retained their pet after receiving assistance (unpublished data). Another limitation of this study is that the comparison group was selected from a spay/neuter clinic and those attending may not be representative of all residents and dogs in the catchment area for the shelter. For example, one would expect dogs coming to a spay/neuter clinic to be younger and healthier than the general population. This selection bias would tend to overestimate the relationship between age or health and intent to relinquish. In addition, the original intent of the study was to include cats but not enough people relinquishing cats were recruited to include these data in the analysis. It is unlikely that these dog findings extrapolate to cats; therefore it will be important in the future to identify risk factors for relinquishment of cats in the South Los Angeles area. This study is also limited by the use of a self-rated measure of perceived stress. There may be recall bias for some respondents. Further, the contribution of specific life events was not assessed and would provide a deeper look into the role of stress in animal relinquishment for future studies. Finally, some questions (for example the question assessing attachment) may be subject to social desirability bias. 

## 5. Conclusions 

These findings reveal a pattern in the home during the time leading up to the decision to relinquish that involves increased perceived stress, as well as emotional attachment to the dog, but an inability to provide the necessary care due to a lack of awareness of resources and an inability to pay for needed services. There were differences in the specific needs of this sample from previous literature. Thus, assessing community needs and providing community-specific alternatives to relinquishment are necessary steps when designing interventions to keep dogs in their homes. Further, in this community, the shelter was the first place relinquishers went. These data suggest that appropriate interventions can take place at the shelter door. And appropriate interventions may provide effective alternate solutions to relinquishment, when those in need of them can access them.
